# Co-creating systems change for mental health: a theory of change approach from the MeHPriC initiative in Lagos, Nigeria

**DOI:** 10.1186/s43058-025-00798-7

**Published:** 2025-11-04

**Authors:** Abiodun Olugbenga Adewuya, Bolanle A. Ola, Olurotimi Coker, Olabisi E. Oladipo, Olushola Olibamoyo, Olayinka Atilola

**Affiliations:** 1 Department of Behavioural Medicine, Lagos State University College of Medicine (LASUCOM), Ikeja, Lagos, Nigeria; 2 Centre for Mental Health Research & Initiative (CEMHRI), Lagos, Nigeria

## Abstract

**Background:**

Integrating mental health services into primary health care (PHC) in low- and middle-income countries (LMICs) is a complex systems-change challenge that requires robust, contextually adapted frameworks. The Mental Health in Primary Care (MeHPriC) initiative in Lagos, Nigeria, aimed to scale up Mental Health Gap Action Programme (mhGAP) based task-sharing for depression, psychosis, and epilepsy. To guide this complex intervention, a participatory Theory of Change (ToC) approach was adopted as a planning, implementation, and governance tool.

**Methods:**

Using a participatory action research design guided by the Consolidated Framework for Implementation Research (CFIR), the MeHPriC ToC was co-created over an 18-month period (2013–2014). The process involved three structured workshops, 36 stakeholder-specific consultations, and four technical working groups with over 150 participants from government, health facilities, and communities. A Community Knowledge, Attitudes, and Practices survey assessed community-level changes in mental health literacy and stigma. A mixed-methods evaluation was conducted (2014–2017) to assess implementation and clinical outcomes using the ToC as an analytical framework, with operational definitions established for key indicators.

**Results:**

The participatory process produced a comprehensive, co-owned ToC map detailing causal pathways, assumptions, and indicators across community, facility, administrative, and state levels. Implementation outcomes included training 320 PHC workers, achieving 69.1% practice adoption and 79.6% fidelity to core protocols. This resulted in a 58.7% increase in mental health consultations and a 60.3% clinical recovery rate for depression. Community stigma remained at 20% post-intervention. A systematic analysis of implementation barriers and facilitators through CFIR domains showed distinct patterns within each domain, such as the need for cultural adaptations, involvement of religious leaders, and the use of hybrid supervision models. Key policy wins included integration of mental health indicators into the state Health Management Information System and establishment of dedicated budget lines for supervision.

**Conclusion:**

A participatory and empirically-refined ToC approach can serve as an effective governance and implementation framework for complex health system interventions in LMIC settings. The MeHPriC experience demonstrates that this methodology guides implementation to achieve positive clinical outcomes while fostering stakeholder alignment necessary for policy integration and long-term sustainability.

**Supplementary Information:**

The online version contains supplementary material available at 10.1186/s43058-025-00798-7.

Contribution to literature

- This study provides the first detailed account of using participatory Theory of Change methodology for mental health integration in West Africa, demonstrating how community-level data collection (*n* = 1,200) can complement facility-based implementation metrics to provide comprehensive system-level insights.
- It shows that systematic application of implementation science frameworks like CFIR can structure complex multi-stakeholder processes, enabling identification of domain-specific barriers and facilitators that inform targeted adaptation strategies in low-resource contexts.
- This paper demonstrates methodological innovations including systematic assumption tracking and hybrid digital supervision models that enable real-time adaptation of complex interventions, offering replicable approaches for managing uncertainty in participatory action research.
- It provides operational definitions and measurement approaches for challenging indicators like community stigma and clinical recovery in resource-constrained settings, contributing to the methodological toolkit for implementation research in global mental health.

## Background

Mental, neurological, and substance use (MNS) disorders represent a significant and growing portion of the global burden of disease, accounting for about 13% of the total. Low- and middle-income countries (LMICs) bear a disproportionate share of this burden due to severe resource limitations [1, 2]. Worldwide, mental health conditions impact more than 970 million people [3]. In low- and middle-income countries (LMICs), more than 75% of individuals affected do not receive formal treatment, with this gap exceeding 90% in certain resource-limited regions [2, 4]. This crisis is particularly acute in sub-Saharan Africa, where under-resourced health systems, a severe shortage of a specialist workforce, and pervasive stigma combine to create formidable barriers to care [5–7]. Nigeria, with a population of over 200 million, exemplifies this challenge. Despite evidence that 20–30% of Nigerian adults will experience a diagnosable mental disorder in their lifetime, fewer than 10% receive any form of evidence-based care [8, 9]. The nation’s mental health system is critically undermined by a shortage of specialists, with fewer than 300 psychiatrists servicing over 200 million people, inadequate financing, poor integration of services at the primary care level, and deep-rooted societal stigma [10].

In response to this global deficit, the World Health Organization (WHO) launched the Mental Health Gap Action Programme (mhGAP) [11]. The programme promotes task-sharing by training non-specialist primary healthcare (PHC) workers to identify and manage priority MNS disorders like depression, psychosis, and epilepsy [12]. This approach facilitates broader health system strengthening by leveraging existing infrastructure and community platforms to reduce barriers related to stigma, cost, and geographic access. Robust evidence confirms that integrating mental health services into primary care can improve clinical outcomes and has demonstrated favourable cost-effectiveness [13]. The mhGAP framework has since been adopted by over 100 countries, with studies in Nigeria showing knowledge gains of up to 82% among PHC workers [14], and research in Kenya reporting practice adoption rates of 72% [15].

However, integrating mental health into PHC is a complex systems intervention involving multiple stakeholders and dynamic implementation contexts. Such complexity necessitates frameworks that can map causal pathways, engage diverse stakeholders, and adapt to emergent challenges [16]. Theory of Change (ToC) has emerged as a key planning and evaluation tool suited to these demands. A ToC provides a structured explanation of how and why an intervention is expected to work, laying out the sequence of preconditions and activities necessary for success while making underlying assumptions explicit [17]. ToC frameworks offer distinct advantages for mental health integration by conceptualizing the interplay between clinical and community-level changes, fostering stakeholder ownership to enhance local relevance, and serving as implementation roadmaps that provide clarity on priorities, sequencing, and evaluation metrics [18, 19]. The utility of this approach has been documented in major global mental health research programmes, including PRIME (Programme for Improving Mental Health Care) [19], which developed district-level mental health care plans across five LMICs, and EMERALD (Emerging Mental Health Systems in LMICs) [20], which strengthened health system responses through implementation research in six LMICs. These efforts underscore the tool’s role in multi-level planning, yet such applications remain sparse in West Africa, where culturally distinct challenges demand locally adapted models.

The Mental Health in Primary Care (MeHPriC) initiative in Lagos, Nigeria, is a flagship effort to integrate mental health services into PHC facilities by scaling up mhGAP-based task-sharing for depression, psychosis, and epilepsy [21]. Launched in 2011, MeHPriC focused on training non-specialist providers, integrating mHealth support, and tailoring stepped-care pathways. Initial training and supervision led to marked improvements in provider knowledge, attitudes, and practice adoption, with a pilot study demonstrating a 60.3% clinical recovery rate from depression in stepped-care versus 18.2% in usual care [21, 22]. However, implementation challenges persisted, including medication supply gaps in 62.4% of rural PHCs, continued community stigma affecting 20% of respondents post-training, and fragmented referral pathways with completion rates below 40% [23]. These findings highlighted the need for a more robust strategic framework to guide scale-up and address systemic barriers.

In response, the MeHPriC consortium adopted a ToC framework to guide its next phase of implementation. The ToC’s enduring applicability is particularly relevant in the West African context, which continues to face a paucity of systematic mental health integration frameworks, making lessons from this long-term initiative valuable for ongoing regional efforts. The aim of this paper is to provide a detailed account of this process and its outcomes. The specific objectives are:

1. To document the participatory development of a Theory of Change framework for mental health integration in Lagos State primary health care.
2. To evaluate implementation outcomes using the developed ToC as a monitoring and evaluation framework.
3. To assess the effectiveness of multi-stakeholder engagement across community, facility, administrative, and state system levels.
4. To provide evidence-based recommendations for ToC replication in similar low- and middle-income country contexts.

## Methods

### Study design and theoretical framework

The ToC for the MeHPriC initiative was developed using a participatory action research (PAR) approach [24]. This methodology was chosen because it emphasizes collaboration between researchers and stakeholders to generate practical, contextually relevant solutions to complex problems. Our PAR process was theoretically underpinned by implementation science frameworks, primarily the Consolidated Framework for Implementation Research (CFIR). CFIR [25] was used to systematically identify and categorize the multilevel determinants of implementation success across its five domains, ensuring a comprehensive assessment of contextual factors. The direct mapping of these domains to our ToC components is detailed in Table 1. To ensure robust reporting of our qualitative methods, the study adhered to the Consolidated Criteria for Reporting Qualitative Research (COREQ) checklist [26].

**Table 1:** Mapping of CFIR domains to theory of change components

CFIR Domain	Mapped ToC Elements
Intervention Characteristics	mhGAP adaptation, job aids, mHealth tools
Inner Setting	PHC infrastructure, supervision structures, leadership engagement
Outer Setting	Community engagement, policy support, stigma reduction
Characteristics of Individuals	Provider confidence, cadre-specific adoption patterns
Implementation Process	Peer supervision, feedback loops, policy and scale-up strategy

### Study setting

Lagos State, Nigeria's most populous and urbanized region with over 20 million residents, presents a compelling context for implementing mental health integration at scale. The state's health system spans diverse urban and rural contexts, with varying facility capacities, infrastructure gaps, and population needs that exemplify the implementation challenges faced by many other LMICs.

### Overall ToC development framework

The ToC development and implementation followed a chronological sequence that reflects the iterative nature of participatory action research. This includes initial development phase (Workshop 1, stakeholder consultations, and working groups, 2013), implementation and data collection phase (January-December 2014), mid-implementation review and refinement (Workshop 2, June 2014), and final sustainability planning (Workshop 3, December 2014). A summary of these engagements, including timelines and participant totals, is provided in Table 2.

**Table 2:** Chronological summary of theory of change development and implementation

Phase	Activity	Timeline	No. of Participants	Relationship to Implementation	Focus	Key Stakeholders
**Phase 1: ToC Development**	Workshop 1	June 2013	50	Pre-implementation planning	Initial ToC development and pathway mapping	PHC workers, MOH, community leaders, media
	Stakeholder Consultations	April–August 2013	36	Pre-implementation planning	Sector-specific input on feasibility and roles	MOH, PHCB, unions, community gatekeepers
	Working Groups	July 2013	~ 60	Pre-implementation planning	Technical adaptation of protocols and systems	Resource, clinical, training, and governance experts
**Phase 2: Implementation**	Implementation Launch	January 2014	320 PHC workers trained	Active service delivery begins	mhGAP training and service implementation	PHC providers, supervisors, communities
**Phase 3: Mid-Implementation Review**	Workshop 2	June 2014	48	Mid-implementation (6 months)	Validation using pilot data and ToC refinement	Program staff, supervisors, policymakers
**Phase 4: Implementation Continuation**	Ongoing Implementation	July–December 2014	All stakeholders	Continued service delivery with adaptations	Refined protocols and supervision	All implementation actors
**Phase 5: Sustainability Planning**	Workshop 3	December 2014	52	End of implementation phase	Planning for scale-up and policy integration	MOH, PHCB, partners, academic collaborators

#### Service user consultation process

While the formal ToC workshops prioritized institutional stakeholders due to the systems-change focus, we conducted supplementary consultations with service users and caregivers between July–September 2013. These included:

- Individual interviews (*n* = 15): In-depth discussions with individuals who had received mental health care at participating PHCs, exploring their experiences with services, barriers to access, and preferences for care delivery
- Caregivers focus groups (*n* = 3, total participants = 22): Sessions with family members of individuals with mental health conditions, examining their perspectives on stigma, treatment adherence, and community acceptance
- Community dialogue sessions (*n* = 4): Public forums in collaboration with community leaders where mental health service users could share experiences anonymously through trained facilitators

These consultations informed community-level pathway development, particularly regarding stigma reduction strategies and service delivery preferences.

### Workshop 1: initial development

The foundational two-day workshop, held in June 2013 with 50 stakeholders, aimed to construct the initial ToC map. Preparatory activities included a situation analysis of existing mental health services, a review of local epidemiological data, and a targeted literature review of integration models. The workshop adapted global ToC methodologies [17, 18] to the Nigerian context, informed by frameworks from programmes like PRIME [19]. Participants were purposively selected to ensure multisectoral representation, including officials from the Lagos State Ministry of Health (LSMoH), frontline PHC providers, union leaders, and community gatekeepers. Invitations were issued through formal channels, such as ministerial letters and coordination via the Primary Health Care Board (PHCB), to secure institutional buy-in. A detailed breakdown of participants by constituent group is provided in Supplementary Table S1. During the workshop, participatory tools like visual mapping, small group discussions, and the use of culturally adapted metaphors were employed to facilitate backward mapping from long-term goals to immediate preconditions.

### Stakeholder-specific meetings and consultations

To deepen the insights from the main workshop, 36 stakeholder-specific consultations were conducted between April and August 2013. This process blended continuity with new perspectives; approximately 60% of participants had also attended Workshop 1, while 40% were new stakeholders identified through snowball sampling. The selection process used a maximum variation strategy to ensure a wide range of views within each stakeholder category. The consultations comprised 28 individual interviews and 8 small-group sessions, all conducted in-person at stakeholders workplaces to maximize convenience and contextual relevance. Individual interviews (60–90 min each) were conducted with senior officials including LSMoH directors (*n* = 5), PHCB coordinators (*n* = 3), facility managers (*n* = 8), union representatives (*n* = 4), media professionals (*n* = 2), religious leaders (*n* = 4), and local government officials (*n* = 2). Small group sessions (2–5 participants each) involved frontline PHC workers organized by cadre (nurses, CHEWs, doctors) and community leaders by geographic area (urban/rural). This mixed format optimized both depth of individual perspectives and group consensus-building within stakeholder categories. Facilitators used semi-structured guides with standardized core questions, but also incorporated role-specific probes to explore topics like policy feasibility with government officials and stigma reduction with community leaders. The complete interview guides are available in Supplementary Appendix A. Representative perspectives from these consultations, illustrating the range of stakeholder views and their evolution throughout the ToC development process, are presented in Supplementary Table S2.

### Thematic working group sessions

In July 2013, four technical working groups (10–15 participants each) were convened to develop specific ToC components related to Resources, Clinical Content, Training, and Implementation Support. Participants self-selected into groups based on their expertise, with facilitators ensuring a balanced mix of policymakers, practitioners, and technical experts in each group. The outputs from these groups, including draft clinical protocols, training manuals, and supervision plans, were evidence-based and contextually grounded, and were subsequently integrated into the evolving ToC framework.

### Implementation phase and data collection

Following the initial ToC development (Workshops 1, consultations, and working groups), the implementation phase began in January 2014. Data collection occurred continuously throughout this phase using the mixed-methods design described below.

- Pre-implementation phase (April-December 2013): Baseline Knowledge, Attitudes, and Practices (KAP) surveys (*n* = 265) were administered to all PHC workers in May–June 2013, prior to Workshop 1, to establish baseline mental health knowledge and practice patterns. Organizational Readiness to Change Assessment (ORCA) surveys (*n* = 40) were conducted at facility level in June-July 2013 to measure institutional preparedness before ToC workshops. Community-Level Assessment: In addition to provider-level data collection, a Community Knowledge, Attitudes, and Practices (KAP) survey was administered to assess broader community-level changes in mental health literacy and stigma. The survey employed a stratified random sampling approach across 24 communities in participating PHC catchment areas, yielding a sample of 1,200 community members at baseline (May–June 2013) and follow-up (January-March 2015). The survey utilized culturally adapted instruments to assess mental health knowledge, stigmatizing attitudes, help-seeking preferences, and exposure to project activities. This community-level data collection complemented facility-based indicators and provided essential context for interpreting service utilization patterns and sustainability outcomes.
- Implementation phase (January-December 2014): Post-training KAP surveys (*n* = 265) were re-administered 2–4 weeks after mhGAP training completion (February-April 2014) to assess immediate knowledge changes. Monthly service metrics were collected continuously from facility Health Management Information System (HMIS) registers beginning January 2014. Early data from this period (January-June 2014) informed Workshop 2 refinements. Mid-implementation ORCA assessments (*n* = 40) were repeated at 6 months post-training (July–August 2014)
- Evaluation phase (January-June 2015): Six-month follow-up KAP surveys (*n* = 265) provided final assessment of sustained knowledge retention and practice adoption. Structured clients exit interviews (*n* = 500) were conducted over 6 months (January-June 2015) to capture service user experience. Qualitative data were drawn from 12 focus group discussions (FGDs) with PHC workers and 10 key informant Interviews (KIIs) with managers and trainers conducted between March–May 2015, full workshop transcripts, and detailed field notes.

Our data collection approach was designed to enable systematic validation of ToC assumptions through empirical evidence. The sequential timing (Supplementary Table S3) allowed quantitative findings from KAP surveys and ORCA assessments to inform qualitative inquiry design, while comprehensive mapping of indicators to data sources (Supplementary Table S4) ensured robust measurement of outcomes across all system levels. This approach facilitated the triangulation strategy detailed in Supplementary Table S5, enabling identification of convergent and contradictory findings that informed ToC refinements.

### Workshop 2: Mid implementation refinement

At the midpoint of the implementation phase (June 2014), Workshop 2 convened 48 participants to review preliminary implementation data and refine the ToC based on early experiences. Preparatory analysis involved a thorough review of pilot implementation data collected during the first six months of service delivery (January-June 2014). These preliminary data, comprising HMIS usage rates, training completion statistics, and qualitative feedback from PHC staff, were distinct from the previously published pilot studies referenced in the Background section [21, 22]. The implementation monitoring data highlighted early challenges including medication stock-outs and referral bottlenecks and have been partially incorporated into subsequent publications from the broader MeHPriC initiative. During the workshop, these findings were presented to stakeholders, who then worked to validate core assumptions and adapt implementation strategies. It was here that mHealth strategies were formally prioritized through a structured group consensus process, with participants scoring each option on a 1–5 scale across feasibility, familiarity, and cost-effectiveness criteria.

### Workshop 3: scale-up and sustainability

The final workshop, conducted in December 2014 with 52 participants, focused on institutionalization and state-wide scale-up. Preparatory steps included detailed outcome and cost-effectiveness reviews of the pilot phase and an analysis of alignment with upcoming policy windows. The workshop resulted in finalized scale-up plans, stakeholder commitment declarations, and policy briefs to support long-term sustainability.

### Data analysis methods

#### Qualitative analysis

We employed framework analysis [27] for our qualitative data. This systematic approach involved five stages: familiarization with the data; identifying a thematic framework (using CFIR domains as a priori deductive codes); indexing the data against the framework while allowing inductive sub-themes to emerge; charting the data into framework matrices; and mapping and interpretation to explain findings. The analysis was conducted by a team of three trained researchers using NVivo 12 software. To ensure reliability, 20% of transcripts were independently coded, achieving high inter-rater reliability (κ ≥ 0.8), with discrepancies resolved through weekly consensus meetings. An external implementation science expert audited a sample of the coded data to verify methodological rigor.

#### Quantitative analysis

Quantitative data were analyzed using SPSS v25. Descriptive statistics (frequencies, percentages, means) were used to summarize sample characteristics and key outcomes like consultation and recovery rates. Inferential statistics were used to assess change; paired t-tests compared pre- and post-training knowledge scores, while chi-square tests were used for categorical comparisons between urban and rural sites. Odds ratios were calculated to determine the impact of the intervention on provider self-efficacy.

The sequential data collection design enabled validation of ToC assumptions through empirical evidence. For example, when 6-month KAP surveys revealed practice adoption rates of 69.1% (below the projected 85% target), this finding prompted targeted exploration in subsequent FGDs to identify implementation barriers. Similarly, urban–rural disparities identified in quantitative metrics (71.2% vs. 65.8% adoption rates) led to geographic-specific probes in qualitative interviews, informing ToC refinements during Workshop 2.

#### Mixed-methods integration

We used a sequential explanatory design [28] to integrate our data streams. In *Phase 1*, quantitative analysis identified key implementation patterns and unexpected findings. In *Phase 2*, these findings informed the qualitative inquiry; for example, interview guides for FGDs were adapted to explore why high provider knowledge scores did not uniformly translate into practice change. In *Phase 3*, a convergent parallel approach was used, with integration matrices comparing quantitative results and qualitative themes side-by-side. This allowed for triangulation to generate meta-inferences that provided a more comprehensive explanation of the implementation process. Our systematic triangulation strategy, designed to identify convergent, complementary, and contradictory findings across data sources, is outlined in Supplementary Table S5.

#### Operational definitions for primary indicators

##### Stigma measurement

Community stigma was operationalized using a 12-item scale adapted from Corrigan et al.'s Attribution Questionnaire [29], measuring social distance preferences and discriminatory beliefs about mental illness. Items included statements such as "*I would avoid people with mental illness*" and "*Mental illness is caused by spiritual problems*" (5-point Likert scale: 1 = strongly disagree to 5 = strongly agree). Respondents scoring above the 75th percentile on negative attribution items were classified as exhibiting high stigma.

##### Clinical recovery definition

Depression recovery was operationally defined using a combination of symptom reduction and functional improvement criteria. Primary assessment utilized clinician-rated improvement based on structured clinical interviews at 3, 6, and 12-month intervals, supplemented by the Patient Health Questionnaire-9 (PHQ-9) [30, 31] where feasible. Recovery was defined as transition from moderate/severe symptoms at baseline to mild/minimal symptoms sustained for at least 4 weeks, coupled with functional improvement assessed through provider-rated return to usual activities.

##### Provider practice adoption

Measured through direct observation checklists during quarterly supervision visits, assessing adherence to key mhGAP protocol elements including screening procedures, diagnostic approaches, treatment planning, and documentation practices. Adoption rate reflects the proportion of observed consultations demonstrating adherence to at least 80% of protocol elements.

### Quality assurance and ethical considerations

Rigor was ensured through systematic data triangulation, stakeholder validation of the ToC pathways, and expert review. Member checking was conducted with 10 stakeholders who reviewed a summary of findings to ensure the interpretations were credible and representative. All analytical decisions were documented in a detailed audit trail. Ethical clearance was obtained from the Lagos State Health Research Ethics Committee (LSHREC/12/2013/313). Written informed consent, including for audio recordings, was secured from all participants.

## Results

### Part 1: ToC development process and outcomes (2013–2014)

#### Workshop 1: initial development outcomes

The initial ToC workshop successfully established a strong foundation of cross-sectoral legitimacy and ownership. The engagement of 50 stakeholders, reinforced by 36 subsequent consultations, secured critical buy-in. The breadth of engagement is evidenced by the diversity of participants (Supplementary Table S1) and the range of perspectives captured during the process (Supplementary Table S2). This process yielded tangible commitments, including a 10% co-funding pledge from the LSMoH and an endorsement from the PHCB to integrate mental health indicators into the HMIS. Professional associations and health worker unions aligned on proposed task-sharing roles, a crucial step for implementation. As one workshop participant noted, (Supplementary Table S2), the process fundamentally shifted perspectives:

*"The ToC helped us see mental health not as an add-on, but as integral to PHC, it changed our whole thinking."*

**Fig. 1:**
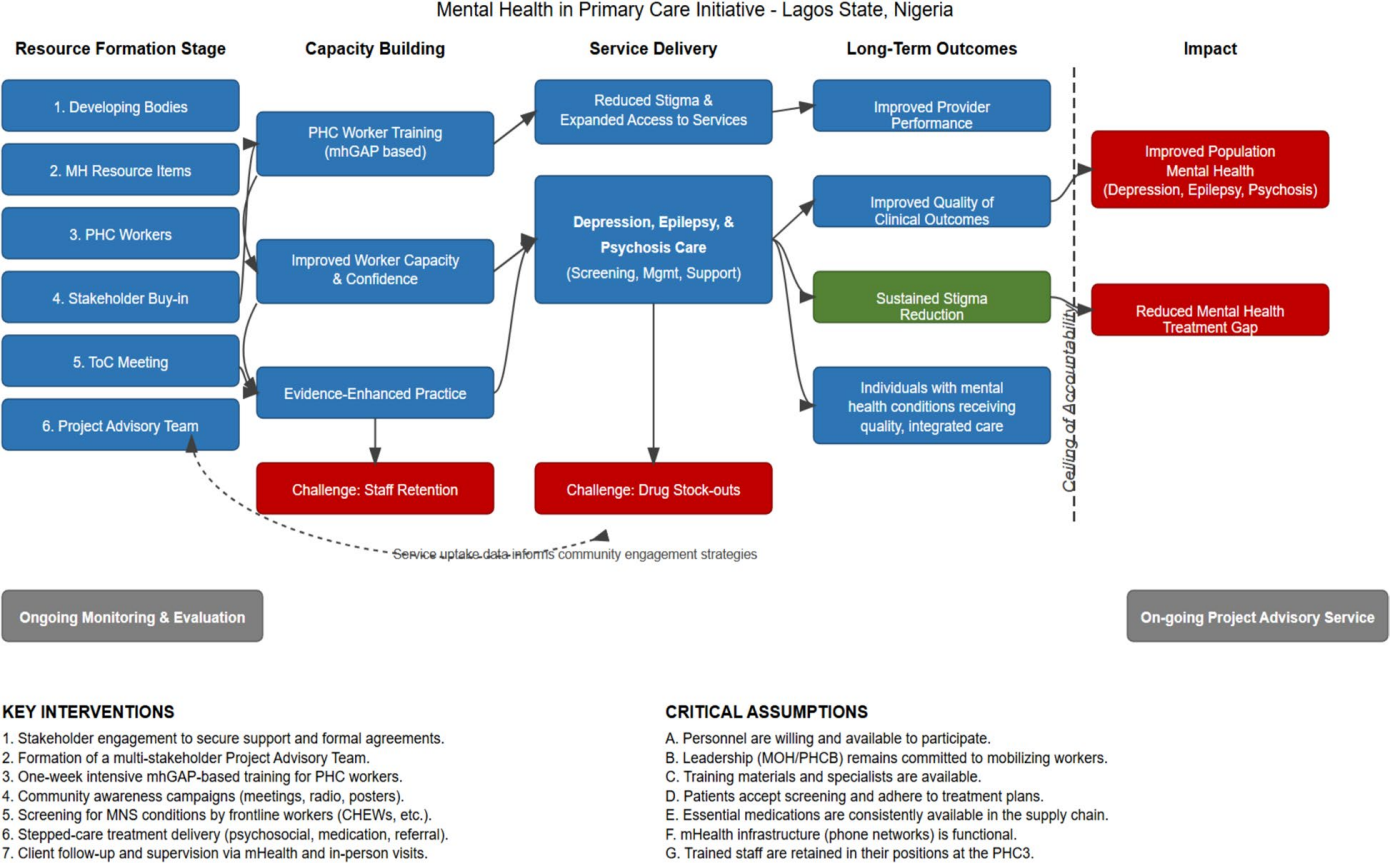
Mehpric theory of change map

The initial ToC map, developed collaboratively during this workshop (Fig. 1), was structured across four interlinked levels: community, health facility, administrative, and state systems. It incorporated cross-cutting components and a "*ceiling of accountability*" to distinguish outcomes directly attributable to the MeHPriC initiative from those contingent on broader systemic factors beyond the project's direct control.

The thematic working groups provided the operational details for the ToC. The Resources Group established infrastructure benchmarks, while the Clinical Intervention Group localized mhGAP protocols, assigning specific roles to different cadres and developing culturally adapted training materials in Yoruba. The Training and Supervision Group designed the core capacity-building program, and the Implementation Support Group developed strategies for governance and HMIS integration (Table 3).

**Table 3:** Key Themes from Working Group Sessions and mhGAP Adaptations

Working Group	Key Outputs
Resources	Infrastructure plans, MOH co-funding, staffing projections
Clinical Content	Adapted mhGAP protocols, cadre-specific roles, Yoruba training materials
Training & Supervision	1-week training curriculum, 90% competency target, hybrid supervision model
Implementation Support	Drug supply systems, HMIS integration, policy alignment strategies

### ToC Map components and causal pathways

The ToC framework detailed four primary causal pathways, each with specific activities, outcomes, and underlying assumptions. A summary of these pathways is presented in Table 4.

- *Community-Level Pathway***:** This pathway focused on increasing mental health literacy and reducing stigma through targeted campaigns and engagement with community gatekeepers.
- *Health Facility-Level Pathway***:** Interventions at the facility level were anchored in mhGAP training and the implementation of stepped-care protocols. A key precondition was ensuring the availability of trained staff and essential psychotropic medications.
- *Administrative-Level Pathway***:** This involved establishing local government coordination, deploying routine supervision, and operationalizing digital peer support to ensure fidelity and motivation.
- *State-Level Pathway:* At the highest level, the initiative worked to embed mental health within policy frameworks, secure dedicated budget lines, and establish functional referral linkages with specialist services.

**Table 4:** Summary of ToC pathways, core activities, outcomes, and assumptions

Pathway	Core Activities	Short- and Medium-Term Outcomes	Key Assumptions
Community Engagement	Media campaigns, religious leader sensitization, dialogues with traditional healers	Improved mental health literacy, reduced stigma, increased demand for PHC-based services	Community leaders are willing partners; beliefs can shift through sustained efforts
Health Facility Capacity	mhGAP training, job aids distribution, implementation of stepped-care model	Knowledge gain (59.3% to 81.0%), 69.1% protocol adoption, 60.3% recovery for depression	Trained staff remain; drug supply is consistent; task clarity improves adoption
Supervision and Feedback	Quarterly visits, peer support via WhatsApp, feedback from trainers	79.6% fidelity to mhGAP protocols; staff motivation and consistency improved	Supervisors retained; staff responsive to hybrid models; peer learning is acceptable
Systems Integration	HMIS revision, policy briefs, budgeting, curriculum incorporation for nurses/CHEWs	Services institutionalized, scale-up to 60 PHCs planned, indicators embedded in state systems	Cross-sector collaboration remains strong; political will supports mental health reform

### Workshop 2: refinement and validation outcomes

In June 2014, 48 participants reconvened to review eight months of pilot implementation data. The review confirmed the promise of the intervention but also exposed critical implementation bottlenecks. Drawing on this evidence, stakeholders introduced several data-driven refinements. To address medication shortages, supply chains were diversified through partnerships with private pharmacies. The supervision model was adapted from infrequent site visits to a hybrid approach combining in-person check-ins with ongoing WhatsApp peer support. Referral systems were streamlined by appointing facility-level focal persons and introducing bidirectional feedback loops to improve coordination. These adaptations demonstrated the ToC's function as a dynamic tool for learning and responsive problem-solving.

### Workshop 3: Scale-up and sustainability outcomes

The final workshop in December 2014, attended by 52 stakeholders, focused on institutionalization. The process culminated in a formal commitment from the MOH to scale MeHPriC to 60 additional PHCs. Key policy wins included the formal incorporation of mental health indicators into the state HMIS and the establishment of a dedicated mental health budget line for supervision. The finalized ToC map reflected this accumulated learning, embedding measurable targets for the scale-up phase, including a 70% provider adoption rate and a 50% reduction in the local mental health treatment gap.

## Part 2: implementation evaluation and sustainability (2014–2017)

### Implementation outcomes and variations

During the implementation phase, 320 PHC workers were trained, with 87% achieving competency benchmarks. Across participating facilities, 1,890 clients were screened for mental health conditions, leading to a 15% depression diagnosis rate. The intervention achieved a 60.3% clinical recovery rate for depression, and treatment adherence reached 78.1%. Overall, mental health consultations recorded in the HMIS increased by 58.7% from baseline. A detailed breakdown of these outcomes by system level is provided in Table 5.

**Table 5:** Implementation outcomes by system level

Level	Key Indicators
Community	65.3% increase in urban consultations; stigma remained at 20% post-intervention
Facility	Knowledge improvement (59.3% to 81.0%), 60.3% recovery rate, 69.1% protocol uptake
Administrative	Quarterly supervision (81.9%), 58.7% increase in HMIS mental health data entries
State	15 PHCs integrated; policy budget lines created; referral coordination initiated

Provider self-efficacy improved significantly (OR = 1.517), and practice adoption among trained providers was 69.1%, with 79.6% fidelity to core mhGAP protocols (Table 6). The impact on providers' confidence was a recurring theme. One PHC nurse explained:

*"Before training, I was afraid to ask about depression. Now I see it as part of comprehensive care, like checking blood pressure."*

**Table 6:** Summary of key implementation outcomes from the MeHPriC initiative

Outcome Domain	Indicator	Result
**Provider Capacity Building**	PHC workers trained	320
	Competency benchmark reached post-training	87%
	Provider practice adoption of new protocols	69.1%
**Service Delivery**	Increase in mental health consultations	+ 58.7%
	Clients screened for mental health conditions	1,890
	Fidelity to core mhGAP protocols	79.6%
**Clinical Outcomes**	Clinical recovery rate (depression)	60.3%
	Patient treatment adherence at 12 months	78.1%

Performance, however, varied significantly by geography. Urban PHCs consistently outperformed their rural counterparts across key indicators, including increases in consultations and rates of provider adoption. These disparities were linked to underlying structural factors, with urban sites reporting fewer medication stock-outs and more consistent supervisory engagement. Rural PHCs faced additional barriers related to transport for supervisors, lower patient literacy, and a stronger cultural reliance on traditional and faith-based care.

Performance also varied by provider cadre. Nurses demonstrated the highest rates of protocol adoption (78%) and were frequently cited as champions for stigma reduction within their facilities. Doctors achieved high diagnostic accuracy but sometimes expressed initial skepticism toward task-sharing. Community Health Extension Workers (CHEWs) proved highly effective in community outreach and case identification, though they required ongoing support to maintain clinical competencies. These quantitative outcomes were systematically tracked through multiple data sources (Supplementary Table S4), enabling robust triangulation with qualitative findings (Supplementary Table S5). Detailed statistical analyses supporting these implementation outcomes, including test statistics, p-values, and effect sizes where applicable, are provided in Supplementary Table S6.

### Barriers, facilitators, and sustainability indicators

Supplementary Table S7 provides a comprehensive mapping of all barriers, facilitators, and sustainability indicators to CFIR domains. Implementation outcomes were systematically analyzed using the Consolidated Framework for Implementation Research (CFIR) domains to identify key determinants of success and sustainability. This analysis revealed domain-specific patterns that inform future scale-up efforts and demonstrate how the participatory ToC process addressed implementation challenges across multiple system levels.

#### Intervention characteristics

The mhGAP-based intervention required substantial adaptation to fit local contexts, creating both facilitators and barriers to implementation. The cultural localization of training materials into Yoruba proved crucial for provider adoption, with 78% of nurses reporting improved confidence when using local language resources. The stepped-care model's modular design allowed flexible implementation across diverse facility types, enabling providers to adapt protocols to their specific contexts. However, initial mhGAP protocols required extensive modification for local contexts, particularly around medication dosing and referral pathways. Some providers reported difficulty managing the complexity of multi-condition protocols, though this improved with experience and ongoing supervision.

#### Outer setting

External contextual factors presented both significant challenges and unexpected opportunities. Community stigma remained a persistent barrier, with 20% of community members post-intervention still endorsing discriminatory attitudes toward mental illness. Chronic medication stock-outs affected 62.4% of rural PHCs, representing system-level failures beyond the project's direct control.

Nevertheless, engagement with community gatekeepers proved transformational. Religious leaders emerged as crucial allies, with communities showing 40% higher service utilization where faith-based leaders actively supported the initiative. As one community leader reflected:

*"When our religious leaders started talking about mental health as health, not spirit problems, that is when people began to listen and trust the clinics."*

State-level policy alignment created favorable conditions for budget integration and HMIS incorporation, demonstrating the importance of political will for sustainability.

#### Inner setting

Facility-level factors significantly influenced implementation success. Strong leadership from the Lagos State Ministry of Health provided crucial institutional support, including a 10% co-funding commitment and formal policy endorsement. The utilization of existing PHC infrastructure proved cost-effective, requiring minimal additional investment for service integration. However, facility-level resource constraints created ongoing challenges. Inconsistent electricity and limited private consultation spaces hampered confidential mental health consultations. Rural facilities faced additional barriers related to transportation costs for supervision visits and lower baseline infrastructure capacity, contributing to the observed urban–rural performance disparities.

#### Characteristics of individuals

Provider-level factors showed significant variation by cadre and individual characteristics. The intervention significantly improved provider self-efficacy (OR = 1.517, 95% CI: 1.23–1.87, *p* < 0.001), with nurses emerging as particularly effective champions for stigma reduction within facilities. As one PHC nurse explained:

*"Before training, I was afraid to ask about depression. Now I see it as part of comprehensive care, like checking blood pressure. The job aids give me the confidence to know what to do next."*

Provider confidence varied substantially by cadre, with CHEWs requiring more intensive ongoing support to maintain clinical competencies despite their effectiveness in community outreach. Initial provider skepticism, particularly among doctors, created temporary implementation barriers. However, attitudes shifted with experience, as one PHC doctor noted:

*"At first I was skeptical about nurses managing depression. But the protocols are clear, the supervision is good, and honestly, they are sometimes better at the counseling than we doctors are."*

#### Implementation process

Process-related factors proved critical for sustained implementation success. The participatory ToC development process fostered unprecedented stakeholder ownership, contributing to 85% of facilities maintaining active service delivery six months post-implementation. The innovative WhatsApp-based peer supervision model overcame logistical barriers while maintaining support quality. As one PHCB supervisor explained:

*"The WhatsApp group was a game-changer. Instead of waiting three months for a site visit, a nurse can ask a question about medication and get an answer from a peer or a specialist in minutes. It solved so many small problems before they became big ones."*

However, referral system fragmentation resulted in completion rates below 40%, indicating persistent gaps in care coordination. Some facilities struggled with data reporting requirements, necessitating additional technical assistance. The impact on service users was nonetheless evident, as one client noted:

*"Before, when I went to the clinic feeling sad and tired all the time, they would just give me vitamins. Now the nurse sits with me, asks real questions, and I got medicine that actually helped."*

The broader policy implications were recognized by local government officials, with one official stating:

*"We have seen the data—more people getting help, families staying together, people returning to work. This is not just health care, this is community development."*

Sustainability was assessed six months after the intensive implementation phase concluded, revealing that 85% of initial PHC facilities continued active mental health service delivery, 70% of trained supervisors remained engaged, and 60% of districts sustained community engagement mechanisms. The institutionalization of mental health indicators within the state HMIS and protection of dedicated budget lines demonstrated successful integration into core health system functions.

## Discussion

### Methodological contributions and ToC process innovations

The MeHPriC initiative contributes to the growing evidence base on applying ToC methodologies in complex health systems, particularly within the under-resourced contexts of sub-Saharan Africa. Our multi-stage process, spanning initial development, empirical refinement, and scale-up planning, allowed the ToC to function not as a static blueprint, but as a dynamic tool for adaptive implementation [19]. This iterative approach aligns with calls for implementation strategies that can respond to the emergent and often unpredictable nature of systems change [32].

A key innovation was the breadth of stakeholder engagement, which moved beyond technical experts to include community gatekeepers, religious leaders, and media professionals. The unusually broad stakeholder engagement in MeHPriC (Supplementary Table S1) proved crucial for fostering shared ownership, as evidenced by stakeholder reflections on the process (Supplementary Table S2). This participatory process appears to have been crucial for building the shared ownership and the political will necessary to overcome implementation barriers, a finding consistent with literature emphasizing the importance of co-creation in settings where top-down approaches often fail [17]. Furthermore, the structured use of thematic working groups provided technical depth while maintaining participatory integrity, ensuring that outputs like clinical protocols and supervision models were both evidence-based and contextually resonant. The systematic tracking of assumptions, such as staff retention and medication availability, and the integration of risk mitigation strategies directly into the implementation plan represents a methodological advance, offering a model for managing uncertainty in real-world settings. This methodological innovation, supported by our systematic data collection timeline (Supplementary Table S3) and triangulation strategy (Supplementary Table S5), enabled real-time adaptation that proved critical for sustained implementation.

### Implementation science insights and evidence integration

Our study affirms the utility of the CFIR [25] in structuring a participatory ToC process. By mapping our activities and outcomes to CFIR domains, we could systematically analyze the determinants of implementation success. For instance, the adaptation of mhGAP materials into Yoruba (Intervention Characteristics) and the strong leadership from the MOH (Inner Setting) were clear facilitators. Conversely, persistent community stigma (Outer Setting) and variable provider confidence (Characteristics of Individuals) were significant barriers that required targeted strategies. Our experience supports recent work suggesting that CFIR can be effectively optimized for LMIC contexts, though it requires careful adaptation to local cultural and systemic realities [33].

#### Comparison with Other ToC Applications

Our findings offer a valuable point of comparison with other large-scale mental health implementation programs. The provider adoption rate in MeHPriC (69.1%) compares favourably with experiences from similar programs: higher than reported in some PRIME sites in South Africa (23–50% adoption depending on sites) [34] but lower than Ethiopia (81.7%) [35]. This variation suggests that while mhGAP training is effective, local contextual factors heavily influence its translation into practice, supporting recent evidence that core intervention components may be universal, but implementation strategies must be highly tailored [36].

#### Participatory ToC development

Our multi-stakeholder approach aligns with emerging applications of ToC methodology in other LMIC mental health contexts. Recent work in Ghana using ToC for district mental health planning [37] similarly demonstrated the value of participatory approaches in developing contextually relevant mental health care plans, though their focus was on district-level implementation rather than the facility-level integration emphasized in MeHPriC.

#### Service user engagement gap

A critical limitation identified in our ToC process was the insufficient meaningful engagement of service users and caregivers during framework development. This gap is increasingly recognized as problematic in LMIC mental health implementation. Recent work from Ethiopia specifically developing a ToC for service user and caregiver involvement in mental health system strengthening [38] highlights the importance of embedding service user perspectives from the outset of ToC development, rather than as an afterthought in evaluation phases.

#### Implementation strategies

Our systematic assumption-tracking represents a methodological innovation that extends beyond conventional ToC applications in mental health. While PRIME and AFFIRM programs used ToC frameworks [36], neither described the explicit monitoring of assumptions that we implemented. This approach enabled real-time adaptation—such as diversifying medication supply chains and shifting to hybrid supervision—that proved critical for sustained implementation.

#### Systems integration challenges

Unlike the facility-based supervision models used in Kenya's mhGAP implementation [39], MeHPriC's hybrid digital model (combining in-person visits with WhatsApp support) proved to be a pragmatic adaptation to logistical challenges. However, similar to the EMERALD programme [20], we found that achieving genuine policy integration required sustained advocacy and could not be assumed to follow from successful clinical implementation alone.

### Health system integration and sustainability

The MeHPriC initiative achieved notable success in embedding mental health services within the existing PHC system, evidenced by a 58.7% increase in consultations and the institutionalization of mental health indicators within the state HMIS. This move from standalone project to integrated service addresses a critical challenge identified in a recent systematic review of mental health integration, which emphasized that sustainable integration requires system-level changes beyond clinical training [40].

#### Comparison with integration models

Our integration approach differed from the co-location models commonly implemented in high-income countries. Recent Australian reviews of integrated mental health care emphasize co-located mental health clinicians working with primary care teams [41]. In contrast, MeHPriC achieved integration through task-sharing with existing PHC staff, reflecting resource constraints typical of LMIC settings. This approach aligns more closely with WHO mhGAP principles and demonstrates an alternative pathway to integration.

#### Supply chain challenges

The chronic stock-outs of psychotropic medications in rural facilities (affecting 62.4% of PHCs) remain a major sustainability challenge, reflecting systemic failures common across sub-Saharan Africa [42]. While our adaptive response of partnering with private pharmacies provided temporary solutions, recent evidence [40] identified medication supply chains as a persistent barrier to sustainable mental health integration across LMICs, suggesting that long-term sustainability requires comprehensive health system strengthening, including procurement and distribution reform.

### Urban–rural disparities and equity considerations

The disparities in nearly all metrics between urban and rural facilities underscore the profound impact of structural inequities on implementation success. Rural facilities faced a "triple threat" of lower staffing levels, greater logistical barriers to supervision, and stronger cultural reliance on informal care providers. These findings are consistent with a large body of literature documenting the urban–rural health divide in LMICs [43, 44]. While our ToC-informed adaptations, such as using community volunteer networks and hybrid supervision, offered promising workarounds, they cannot fully compensate for decades of underinvestment in rural health infrastructure. Future scale-up efforts must incorporate a specific equity lens, with differentiated investment strategies that allocate additional resources for logistics, supervision, and culturally nuanced community engagement in rural and underserved areas.

### Cadre-specific patterns and workforce development

The variation in performance across different health worker cadres provides important lessons for task-sharing policies. Nurses emerged as pivotal actors, exhibiting the highest rates of adoption and leadership in stigma reduction. This reinforces their central role in the future of integrated mental health care delivery in Nigeria and elsewhere [14]. The effectiveness of CHEWs in community outreach highlights the value of leveraging this cadre for case identification and mental health promotion, though it also underscores the need for continuous, tailored supervision to support their clinical competencies. The role ambiguity reportedby non-clinical staff suggests that successful task-sharing requires not only training but also clear task delineation, supportive policies, and formal recognition of expanded roles within the health system.

### Limitations and future research directions

This study has several limitations. Its single-state design, while providing depth, may limit the generalizability of findings to other Nigerian states or LMIC contexts with different governance structures and health system capacities. A significant limitation of our ToC development process was the insufficient inclusion of service users and caregivers with lived experience during the initial framework design workshops.

While we conducted post-implementation interviews with service users (Supplementary Table S4), their voices were not adequately represented during the initial framework design, as reflected in our stakeholder demographics (Supplementary Table S1). This reflects broader challenges in LMIC mental health research related to stigma and logistical barriers to patient participation, but it represents a critical gap that future ToC processes must address through dedicated service user advisory groups from the outset [45]. Finally, this study did not include a formal cost-effectiveness analysis, which will be essential for making a comprehensive investment case for national scale-up. Future research should focus on these areas, as well as evaluating the long-term clinical and health system outcomes of the scaled-up intervention.

### Policy and practice implications

Our findings offer several actionable implications for policy and practice. First, the success of nurse- and CHEW-led mental health delivery provides strong evidence to support the formal recognition and regulation of their expanded scopes of practice. Second, the stark urban–rural disparities demand that policymakers adopt differentiated investment strategies to address longstanding inequities in logistics, infrastructure, and supervision. Third, the institutionalization of mental health within HMIS, budgets, and training curricula demonstrates a viable pathway for embedding mental health within the core functions of the PHC system. Finally, the participatory ToC approach itself offers a replicable model for building consensus and technical alignment that could be applied to other complex health system reforms beyond mental health.

## Conclusion

The MeHPriC experience demonstrates that a phased, participatory, and empirically refined Theory of Change can serve as an effective planning and governance tool for integrating mental health into complex PHC systems. Key implementation achievements, including high rates of practice adoption and clinical recovery, validate the framework’s practical utility, while innovations such as systematic assumption tracking and hybrid peer supervision offer replicable strategies for other LMIC contexts. Ultimately, this work suggests that achieving sustainable systems change in global mental health requires more than just technical solutions; it demands inclusive governance, iterative learning, and a deep commitment to contextual responsiveness. When adapted with cultural and systemic sensitivity, the ToC approach holds considerable promise for guiding national scale-up efforts and informing international implementation practice.

## Supplementary Information

Supplementary Material 1.

## Data Availability

All data generated and analyzed during this study and additional materials, such as the finalized Theory of Change map, indicator frameworks, and ToC facilitation tools, are available from the corresponding author on reasonable request.

## Abbreviations

CEMHRI: Centre for Mental Health Research & Initiative
CFIR: Consolidated Framework for Implementation Research
CHEW: Community Health Extension Worker
COREQ: Consolidated Criteria for Reporting Qualitative Research
EMERALD: Emerging Mental Health Systems in LMICs
FGD: Focus Group Discussion
HMIS: Health Management Information System
KAP: Knowledge, Attitudes, and Practices
KII: Key Informant Interview
LMIC: Low- and Middle-Income Country
LSHREC: Lagos State Health Research Ethics Committee
LSMoH: Lagos State Ministry of Health
MeHPriC: Mental Health in Primary Care
mhGAP: Mental Health Gap Action Programme
mhGAP-IG: Mental Health Gap Action Programme – Intervention Guide
MNS: Mental, Neurological, and Substance Use
MOH: Ministry of Health
ORCA: Organizational Readiness to Change Assessment
PAR: Participatory Action Research
PHC: Primary Health Care
PHCB: Primary Health Care Board
PRIME: Programme for Improving Mental Health Care
STaRI: Standards for Reporting Implementation Studies
TIDieR: Template for Intervention Description and Replication
ToC: Theory of Change
WHO: World Health Organization
