# Supplementary material for: Co-creating systems change for mental health: a theory of change approach from the MeHPriC initiative in Lagos, Nigeria
Source: Implement Sci Commun. 2025 Nov 4;6:114. doi: 10.1186/s43058-025-00798-7 (PMC12584243; doi:10.1186/s43058-025-00798-7)

**SUPPLEMENTARY MATERIALS**

**1.0 STaRI Checklist for Submission**

| **Item** | **Description** | **Reported in Manuscript** | **Section/Location** |
| --- | --- | --- | --- |
| 1 | Title identifies the study as an implementation study | Yes | Title page |
| 2 | Abstract structured to reflect both implementation strategy and clinical intervention | Yes | Abstract |
| 3 | Background provides rationale for implementation study | Yes | Section 1.0 BACKGROUND |
| 4 | Describes the context in which implementation occurred | Yes | Section 2.2 Study Setting |
| 5 | Explains the aim(s) of the study | Yes | Section 1.0 BACKGROUND (final paragraph) |
| 6 | Describes the implementation strategy used | Yes | Sections 2.4-2.6, 2.8-2.9 |
| 7 | Describes the clinical intervention being implemented | Yes | Section 3.2 (mhGAP-based care) |
| 8 | Specifies the design of the study | Yes | Section 2.1 Study Design and Theoretical Framework |
| 9 | Details about the population, setting, and implementation actors | Yes | Sections 2.4-2.6; Supplementary Tables S1-S2 |
| 10 | Describes data collection methods | Yes | Section 2.7 Implementation Phase and Data Collection; Supplementary Tables S3-S4 |
| 11 | Describes outcome measures for both implementation and intervention effects | Yes | Section 3.5; Tables 5-6; Section 2.10.4 Operational Definitions |
| 12 | Analysis methods for implementation and clinical outcomes | Yes | Sections 2.10.1, 2.10.2, 2.10.3 |
| 13 | Summarizes the main results relevant to implementation and clinical effectiveness | Yes | Section 3.5 Implementation Outcomes; Section 3.6 CFIR Analysis; Table 6 |
| 14 | Describes any process evaluations conducted | Yes | Sections 3.3, 3.4; Section 3.6 |
| 15 | Reports fidelity to the implementation strategy | Yes | Section 3.5 (79.6% fidelity) |
| 16 | Describes adaptations to the strategy or clinical intervention | Yes | Section 3.3 Workshop 2 refinements; Section 3.6 CFIR domains |
| 17 | Reports any unintended consequences or barriers | Yes | Section 3.6 Barriers, Facilitators, and Sustainability Indicators |
| 18 | Discusses implications for implementation practice or policy | Yes | Section 4.7 Policy and Practice Implications |
| 19 | Reflects on sustainability and scalability | Yes | Section 3.6.5 Implementation Process; Section 4.3 Health System Integration |
| 20 | Discusses strengths and limitations of the study | Yes | Section 4.6 Limitations and Future Research Directions |
| 21 | Funding and ethical considerations described | Yes | Declarations Section |
| 22 | Provides a list of abbreviations | Yes | List of Abbreviations |
| 23 | Refers to reporting checklist used | Yes | Section 2.1 mentions adherence to COREQ; STaRI compliance in supplementary materials |

**2.0 TIDieR Checklist: MeHPriC Intervention**

| **Item** | **TIDieR Checklist Description** | **Response** | **Section/Page** |
| --- | --- | --- | --- |
| **1. Brief name** | Provide the name or acronym of the intervention. | Mental Health in Primary Care (MeHPriC) Initiative | Title Page |
| **2. Why** | Describe the rationale, theory, or goal. | To address mental health treatment gaps in PHC using mhGAP and a participatory ToC framework in Lagos. | Section 1.0 BACKGROUND |
| **3. What (materials)** | Describe materials used. | mhGAP-IG, Yoruba-language training tools, job aids, supervision guides, mHealth tools (WhatsApp, SMS). | Sections 2.4-2.6; Supplementary Table S4 |
| **4. What (procedures)** | Describe procedures, activities, processes. | 3 ToC workshops, 36 stakeholder consultations, 1-week mhGAP training, hybrid supervision, referrals. | Sections 2.4-2.6, 2.8-2.9; Supplementary Table S3 |
| **5. Who provided** | Describe who delivered the intervention. | Trained PHC workers (doctors, nurses, CHEWs), supervised by MOH/LSPHCB staff, supported by CEMHRI. | Sections 2.4-2.6; Supplementary Tables S1-S2 |
| **6. How** | Describe modes of delivery. | In-person workshops, facility-based training, digital (WhatsApp), community outreach, policy dialogues. | Sections 2.4-2.6, 2.7 |
| **7. Where** | Describe locations/settings. | Public primary healthcare centres (urban and rural) in Lagos State, Nigeria. | Section 2.2 Study Setting |
| **8. When and how much** | Describe intensity, duration, frequency. | 1-week training; quarterly in-person supervision supplemented by ongoing WhatsApp peer support. | Sections 2.4-2.6, 2.7; Supplementary Table S3 |
| **9. Tailoring** | Describe if/how the intervention was adapted. | mhGAP protocols localized; Yoruba translations; facility-level role differentiation. | Section 3.3 Workshop 2 refinements; Section 4.2 Implementation Science Insights |
| **10. Modifications** | Changes made during delivery. | Supervision shifted to hybrid model; medication supply diversified via private pharmacies; mHealth tools adapted for offline use. | Section 3.3 Workshop 2 outcomes; Section 3.6 CFIR analysis |
| **11. How well (planned)** | Describe strategies to maintain fidelity. | Standardized materials, competency benchmarks, supervision manuals, WhatsApp-based reporting. | Sections 2.4-2.6, 2.7 Implementation Phase |
| **12. How well (actual)** | Fidelity assessment or deviations from protocol. | 79.6% fidelity to core mhGAP protocols; adaptations noted for medication access and referrals. | Section 3.5 Implementation Outcomes;  Section 3.6 CFIR domains |

**3.0 COREQ Checklist: MeHPriC Qualitative Study**

**Domain 1: Research Team and Reflexivity**

| **Item** | **Description** | **Reported in Manuscript** |
| --- | --- | --- |
| 1 | Interviewer/facilitator identified | Yes -- Section 2.4 (CEMHRI facilitators) |
| 2 | Researcher credentials | Yes -- Author affiliations |
| 3 | Occupation at time of study | Yes -- Author info section |
| 4 | Gender of researchers | Not explicitly stated |
| 5 | Experience and training | Yes -- Formal qualitative research training noted for the 3-person coding team (Section 2.10.1) |
| 6 | Relationship established prior | No prior relationship with most participants noted |
| 7 | Participant knowledge of interviewer | Participants were informed of researcher roles and study goals in opening remarks. |
| 8 | Interviewer characteristics | Acknowledged. A reflexivity statement noting potential bias as intervention developers and evaluators is included in the Discussion (Sections 4.1, 4.6). Addressed through team-based analysis and member checking. |

**Domain 2: Study Design**

| **Item** | **Description** | **Reported in Manuscript** |
| --- | --- | --- |
| 9 | Methodological orientation/theory | Yes -- Participatory action research, Implementation Science, CFIR, and ToC (Section 2.1) |
| 10 | Sampling method | Yes -- Purposive sampling with maximum variation; snowball sampling for additional stakeholders (Section 2.5) |
| 11 | How participants were approached | Yes -- Formal invitations through MOH, PHCB, and institutional channels (Section 2.4) |
| 12 | Sample size | Yes -- 50 (Workshop 1), 36 (Consultations), ~60 (Working Groups), 48 (Workshop 2), 52 (Workshop 3). 12 FGDs, 10 KIIs (Supplementary Table S3) |
| 13 | Non-participation | Not explicitly reported |
| 14 | Data collection setting | Yes -- Workshops at LASUCOM; consultations at government/PHC facilities (Sections 2.4-2.6) |
| 15 | Presence of non-participants | No -- Stakeholder groups were engaged independently |
| 16 | Description of sample | Yes -- Supplementary Table S1 and narrative in Methods |
| 17 | Interview guides provided | Yes -- Mentioned that guides are available in Supplementary Appendix A |
| 18 | Repeat interviews | No repeat interviews were conducted |
| 19 | Audio/visual recording | Yes -- Audio recorded with consent (Sections 2.4-2.5) |
| 20 | Field notes | Yes -- Taken during workshops, FGDs, and consultations |
| 21 | Duration of interviews/workshops | Yes -- 2-day workshops; 1-2 hour consultations (Sections 2.4-2.5) |
| 22 | Data saturation | Yes -- Stated that saturation was determined to be reached after 10 FGDs, with 2 additional groups conducted for confirmation |
| 23 | Transcripts returned for comment | No, but member checking was conducted for validation (Section 2.10.1) |

**Domain 3: Analysis and Findings**

| **Item** | **Description** | **Reported in Manuscript** |
| --- | --- | --- |
| 24 | Number of data coders | Yes -- Three CEMHRI researchers (Section 2.10.1) |
| 25 | Coding tree description | Not explicitly shown, but use of framework analysis with deductive (CFIR) and inductive codes is described (Section 2.10.1) |
| 26 | Derivation of themes | Yes -- Both deductive (from CFIR) and inductive coding used (Section 2.10.1) |
| 27 | Software used | Yes -- NVivo 12 (Section 2.10.1) |
| 28 | Participant feedback on findings | Yes -- Member checking conducted with 10 stakeholders to validate findings (Section 2.10.1) |
| 29 | Quotations presented | Yes -- Illustrative excerpts from workshops and FGDs included in Results section and Supplementary Table S2 |
| 30 | Data-finding consistency | Yes -- Themes aligned with data and ToC revisions |
| 31 | Clarity of major themes | Yes -- Clearly presented in Results sections 3.1-3.6, structured around CFIR domains |
| 32 | Clarity of minor themes | Yes -- Urban-rural disparities and cadre-specific variations reported (Section 3.5) |

**4.0 Supplementary Table S1: Stakeholder Demographics for ToC Workshop 1**

| **Stakeholder Category** | **Constituent Groups** | **Number of Participants** |
| --- | --- | --- |
| **Government & Policy** | Lagos State Ministry of Health (LSMoH) Officials | 8 |
| **Healthcare Providers** | Primary Health Care (PHC) Workers: | 15 |
|  | - Doctors | (5) |
|  | - Nurses | (7) |
|  | - CHEWs | (3) |
| **Community Representatives** | Community & Religious Leaders | 12 |
| **Civil Society** | Health Worker Union Representatives | 8 |
|  | Media Professionals (Press Associations) | 4 |
| **Research & Academia** | CEMHRI Researchers / Facilitators | 3 |
| **Total** |  | **50** |

*Note: The overall participation rate for invitations was 83%.*

**5.0 Supplementary Table S2: Illustrative Stakeholder Quotes**

| **Stakeholder Type** | **Quote** | **Context** |
| --- | --- | --- |
| **Workshop Participant (MOH Official)** | *"The ToC helped us see mental health not as an add-on, but as integral to PHC—it changed our whole thinking. For the first time, we had a map that everyone, from the ministry to the clinic, could agree on."* | Workshop 1 feedback session |
| **PHC Nurse (Urban Facility)** | *"Before training, I was afraid to ask about depression. Now I see it as part of comprehensive care, like checking blood pressure. The job aids give me the confidence to know what to do next."* | 6-month follow-up FGD |
| **Community Leader (Rural Area)** | *"When our religious leaders started talking about mental health as health, not spirit problems, that is when people began to listen and trust the clinics. The partnership was essential."* | Community consultation session |
| **PHCB Supervisor** | *"The WhatsApp group was a game-changer. Instead of waiting three months for a site visit, a nurse can ask a question about medication and get an answer from a peer or a specialist in minutes. It solved so many small problems before they became big ones."* | Key informant interview |
| **CHW (Rural Facility)** | *"People used to be afraid when we talked about mental health. But after the community meetings with the imam and the pastor together, families started bringing their relatives to the clinic instead of hiding them."* | Provider focus group discussion |
| **PHC Doctor (Urban Facility)** | *"At first I was skeptical about nurses managing depression. But the protocols are clear, the supervision is good, and honestly, they are sometimes better at the counseling than we doctors are."* | Post-implementation interview |
| **Service User** | *"Before, when I went to the clinic feeling sad and tired all the time, they would just give me vitamins. Now the nurse sits with me, asks real questions, and I got medicine that actually helped."* | Client exit interview |
| **Local Government Official** | *"We have seen the data—more people getting help, families staying together, people returning to work. This is not just health care, this is community development."* | Policy dialogue session |

**6.0 Revised Structure for Supplementary Table S3:**

| **Phase** | **Period** | **Data Collection Activity** | **Workshop/**  **Event** | **Data Source** | **Sample/**  **Scope** | **Purpose** |
| --- | --- | --- | --- | --- | --- | --- |
| **Pre-Impleme**  **ntation** | Apr-May 2013 | Baseline situation analysis | *Before Workshop 1* | Document review + stakeholder interviews | 20 key informants | Inform ToC development |
|  | **Jun 2013** | **Workshop 1** | **Initial ToC Development** | Workshop transcripts + field notes | 50 participants | Develop initial ToC framework |
|  | May-Jun 2013 | Baseline PHC worker assessment | *Between consultations* | KAP surveys | n=265 PHC workers | Establish baseline |
|  | Jun-Jul 2013 | Organizational readiness | *Pre-implementation* | ORCA surveys | n=40 facilities | Measure facility preparedness |
| **Impleme-**  **ntation** | Jan-Mar 2014 | mhGAP training delivery | *Implementation begins* | Training registers + assessments | n=320 PHC workers | Capacity building |
|  | Jan 2014-ongoing | Service delivery monitoring | *Continuous data collection* | HMIS data extraction | All facilities | Track service utilization |
|  | **Jun 2014** | **Workshop 2** | **Mid-Implementation Review** | Workshop transcripts + pilot data | 48 participants | Validate and refine ToC |
|  | Jul-Aug 2014 | Mid-implementation assessment | *Post-Workshop 2* | ORCA surveys | n=40 facilities | Track organizational changes |
|  | **Dec 2014** | **Workshop 3** | **Scale-up Planning** | Workshop transcripts | 52 participants | Plan sustainability |
| **Evaluation** | Jan-Jun 2015 | Sustained practice assessment | *Post-implementation* | 6-month follow-up KAP | n=265 PHC workers | Evaluate sustained changes |

**Note:** *This timeline demonstrates the iterative relationship between workshops and data collection, with Workshop 2 serving as a mid-implementation review of preliminary data (Jan-Jun 2014) and Workshop 3 focusing on sustainability planning based on full implementation results."*

**7.0 Supplementary Table S4: Data Source Mapping for Key Indicators**

| **System**  **Level** | **Key Indicator** | **Primary Data Source(s)** | **Collection Method** | **Participants/**  **Data Points** | **Timing** |
| --- | --- | --- | --- | --- | --- |
| **Community** | Mental health literacy improvement | Community KAP surveys | Structured interviews | 1,200 community members | Pre/post intervention |
|  | Stigma reduction | Community KAP surveys; FGDs | Surveys + focus groups | 1,200 surveys + 12 FGDs | Pre/post + ongoing |
|  | Increased demand for services | HMIS consultation data | Routine health records | Aggregated facility data | Monthly |
|  | Community leader engagement | KIIs with leaders; workshop transcripts | Individual interviews | 15 community leaders | Quarterly |
| **Health Facility** | Provider knowledge gain | PHC worker KAP surveys | Pre/post training surveys | n=265 PHC workers | Pre, post, 6-month follow-up |
|  | Provider attitude change | PHC worker KAP surveys | Likert-scale assessments | n=265 PHC workers | Pre, post, 6-month follow-up |
|  | Practice adoption rates | Direct observation checklists; ORCA | Structured observation | PHC staff at n=40 facilities | 6-month intervals |
|  | Clinical recovery rates | Patient clinical records; structured assessments | Electronic health records | 1,890 screened clients | Ongoing during treatment |
|  | Medication availability | Facility stock registers; supervisor reports | Monthly stock counts | All participating facilities | Monthly |
|  | Patient satisfaction | Client exit interviews | Structured interviews | n=500 service users | Over 6-month period |
| **Admini-strative** | Supervision frequency/quality | Supervision logs; WhatsApp group analysis | Digital records + logs | PHCB supervisors + PHC staff | Monthly |
|  | HMIS data completeness | Health information system reports | Electronic database | Aggregated from all sites | Monthly |
|  | Referral completion rates | Referral tracking forms; follow-up calls | Paper + phone tracking | Patient referral cases | Ongoing |
|  | Training completion | Training registers; competency assessments | Administrative records | n=320 trained providers | Post-training |
| **State System** | Policy integration | Policy document analysis; budget reviews | Document review | Government documents | Annual |
|  | Budget allocation | Ministry of Health budget documents | Financial records | MOH budget line items | Annual |
|  | Scale-up commitment | KIIs with MOH officials; policy briefs | Individual interviews | Senior MOH officials | Bi-annual |
|  | Institutional sustainability | ORCA assessments; policy compliance audits | Organizational surveys | System-wide assessment | Annual |

**8.0 Supplementary Table S5: Data Integration and Triangulation Strategy**

| **Research Question** | **Quantitative Data Sources** | **Qualitative Data Sources** | **Integration Method** | **Triangulation Purpose** |
| --- | --- | --- | --- | --- |
| **ToC pathway validity** | KAP survey scores, ORCA assessments | Workshop transcripts, stakeholder interviews | Sequential explanatory | Validate theoretical assumptions with empirical evidence |
| **Implementation fidelity** | Training completion rates, supervision logs | FGDs with providers, KIIs with supervisors | Convergent parallel | Compare reported vs. observed implementation quality |
| **Barriers and facilitators** | Service utilization rates, stock-out frequency | FGDs, stakeholder consultations | Concurrent triangulation | Identify systemic vs. contextual implementation challenges |
| **Sustainability potential** | Budget allocations, policy adoption metrics | KIIs with policymakers, document analysis | Sequential transformative | Assess political will and institutional commitment |
| **Urban-rural disparities** | Disaggregated service statistics, facility assessments | Location-specific FGDs, rural leader interviews | Mixed methods comparison | Understand differential implementation success |
| **User acceptability** | Client exit interview scores, adherence rates | Patient focus groups, community leader interviews | Explanatory sequential | Explain quantitative satisfaction patterns |
| **Cadre-specific adoption** | Training scores by provider type, practice assessments | Cadre-specific FGDs, role-based interviews | Convergent parallel | Understand differential adoption across health worker types |
| **Community engagement effectiveness** | Consultation attendance, media reach metrics | Community leader interviews, religious leader FGDs | Concurrent embedded | Assess depth vs. breadth of community mobilization |

**9.0 Supplementary Table S6:Statistical Analysis of Key Implementation Outcomes**

| **Outcome Measure** | **Baseline/**  **Pre** | **Follow-up/**  **Post** | **Statistical Test** | **Result** | **Effect Size/CI** | **Interpretation** |
| --- | --- | --- | --- | --- | --- | --- |
| Provider Knowledge Score (KAP) | M=59.3 (SD=12.4) | M=81.0 (SD=10.8) | Paired t-test | t(264)=18.7, p<0.001 | Cohen's d=1.89 | Large effect |
| Provider Self-Efficacy | Baseline survey | 6-month follow-up | Logistic regression | OR=2.34, p<0.001 | 95% CI: 1.87-2.93 | Significant improvement |
| Practice Adoption (Urban vs Rural) | Urban: 71.2% | Rural: 65.8% | Chi-square test | χ²=4.23, p=0.040 | Cramer's V=0.16 | Small effect |
| Community Stigma Levels | 35% (baseline) | 20%  (follow-up) | McNemar's test | χ²=12.8, p<0.001 | - | Significant reduction |
| Facility Sustainability | - | 85% at 6 months | Descriptive | - | - | - |

**10. Supplementary Table S7: CFIR Domain Mapping of Implementation Determinants**

| **CFIR Domain** | **Key Barriers** | **Key Facilitators** | **Sustainability Indicators** |
| --- | --- | --- | --- |
| **Intervention Characteristics** | Protocol complexity requiring substantial local adaptation | Cultural adaptations (Yoruba materials), flexible stepped-care design | 78% provider confidence in adapted materials |
| **Outer Setting** | Community stigma (20%), medication supply disruptions (62.4% rural PHCs) | Religious leader engagement, state policy alignment | Policy integration achieved, dedicated budget lines established |
| **Inner Setting** | Resource constraints, infrastructure gaps, rural facility challenges | Strong LSMoH leadership, existing PHC infrastructure utilization | 85% facilities sustained services at 6 months |
| **Characteristics of Individuals** | Cadre-specific confidence variations, initial provider scepticism | Improved self-efficacy (OR=1.517), nurse champions emerged | 70% trained supervisors remained actively engaged |
| **Implementation Process** | Low referral completion (40%), data reporting challenges | Participatory ToC process, WhatsApp supervision innovation | 60% districts maintained community engagement mechanisms |

**11.0 Supplementary Appendices**

**Supplementary Appendix A: Semi-Structured Interview Guide**

**Core Questions (All Stakeholders)**:

1. What are the primary challenges to integrating mental health into primary care in Lagos?
2. What would successful mental health integration look like in 3–5 years, and what measurable changes would be evident?
3. What resources (human, financial, material) are essential for sustainable integration?
4. What key assumptions in the ToC framework might be flawed, and what are the biggest risks to success?
5. What role can your organization or group play in supporting this initiative?

**Role-Specific Probes**:

- **Policymakers (e.g., LSMoH Directors, n=5)**:
  - What policy changes are needed to support task-sharing for mental health?
  - How can MeHPriC align with Lagos’s health priorities and budget cycles?
  - What data or evidence would justify state-wide scale-up?
  - How can competing health priorities (e.g., maternal health) be balanced?
- **PHC Providers (e.g., Nurses, Doctors, CHEWs, n=15)**:
  - What barriers do you face in adopting mhGAP protocols in daily practice?
  - How can training and supervision be tailored to your cadre’s needs?
  - What support would enhance your confidence in managing mental health cases?
  - How do patients’ cultural beliefs impact your service delivery?
- **Community Leaders (e.g., Religious, Traditional Leaders, n=12)**:
  - What are common community beliefs about mental illness, and how can stigma be addressed?
  - Who are trusted health information sources in your community, and how can they be engaged?
  - What would encourage community members to seek PHC services over traditional healers?
  - How can community campaigns align with cultural or religious values?

**Supplementary Appendix B: Community KAP Survey Questionnaires**

Adapted from the Community Attitudes toward Mental Illness (CAMI) scale, the 25-item questionnaire uses a 5-point Likert scale (1=Strongly Disagree, 5=Strongly Agree) to measure stigma and literacy,

**Pre- and Post-Intervention Questionnaire (Identical Items)**:
**Section 1: Sociodemographic Data**

1. Age (years): ______
2. Gender: □ Male □ Female □ Other: ______
3. Marital Status: □ Single □ Married □ Divorced/Separated □ Widowed
4. Educational Attainment: □ None □ Primary □ Secondary □ Tertiary
5. Occupation: □ Unemployed □ Student □ Self-employed □ Formal Sector □ Other: ______
6. Religion: □ Christian □ Muslim □ Traditionalist □ Other: ______
7. Ward/Community: __________________

**Section 2: Knowledge about Mental Illness**
*Select “Yes,” “No,” or “Don’t Know”*:

1. Have you ever heard of mental illness? □ Yes □ No □ Don’t Know
2. Can mental illness affect anyone? □ Yes □ No □ Don’t Know
3. Is mental illness the same as madness? □ Yes □ No □ Don’t Know
4. Is mental illness caused by supernatural forces or spirits? □ Yes □ No □ Don’t Know
5. Can mental illness be treated at a health facility? □ Yes □ No □ Don’t Know
6. Are mental illness and epilepsy the same thing? □ Yes □ No □ Don’t Know
7. Do you believe stress or life difficulties can cause mental illness? □ Yes □ No □ Don’t Know

**Section 3: Attitudes/Beliefs Toward People with Mental Illness**
*5-point Likert scale: 1=Strongly Disagree, 5=Strongly Agree*

1. Mental illness is a spiritual punishment rather than a medical condition.
2. People with mental illness are dangerous and should be avoided.
3. Mental illness can be treated effectively in primary care clinics.
4. I would feel ashamed if a family member had a mental illness.
5. Depression is a sign of personal weakness.
6. People with mental illness can recover with proper treatment.
7. I would be unwilling to live next to someone with a mental illness.
8. People with mental illness should be allowed to marry.
9. I would be comfortable working with someone with a mental illness.
10. Mental illness is caused by supernatural forces.
11. PHC workers are trained to manage mental health conditions.
12. People with mental illness should be treated in the community, not locked away.
    13–25. [Additional items on stigma, social distance, and recovery beliefs, tailored to Yoruba idioms, e.g., “feeling heavy in the mind” for depression.]

**Section 4: Practices and Help-Seeking**

1. If a family member developed mental illness, what would you do? (Select all that apply)
   □ Take to health facility □ Take to prayer house/mosque/church □ Consult traditional healer □ Keep at home □ Other: ______
2. Where do people in your community usually go for help when mental illness is suspected?
   □ Health center □ Church/Mosque □ Traditional healer □ Nowhere □ Other: ______
3. Have you attended a talk or campaign about mental health? □ Yes □ No
4. Have you or a family member used PHC mental health services? □ Yes □ No

**Section 5: Sources of Mental Health Information**
*Tick all that apply*:
□ Radio □ TV □ Religious Leader □ Health Worker □ Family/Friends □ Other: ______

**Scoring for Stigma Indicator**: Negative attitude items (1, 2, 4, 5, 7, 10) scored ≥4 (Agree/Strongly Agree) on >75th percentile classify as “high stigma,” with population proportion forming the “% stigma” metric (20% post-intervention).

**Supplementary Appendix C: Depression Clinical Recovery Checklist (PHQ-9/WHODAS Protocol)**

This checklist, used by PHC clinicians to assess depression recovery, details the Patient Health Questionnaire-9 (PHQ-9) and WHO Disability Assessment Schedule 2.0 (WHODAS 2.0) protocols, administered at baseline, 3 months, and 6 months post-treatment.

**Instructions for PHC Clinician**:
Administer PHQ-9 and WHODAS 2.0 at baseline, 3 months, and 6 months. Assess recovery criteria at each interval.

**A. Patient Health Questionnaire-9 (PHQ-9)**:
*Assess symptoms over the past 2 weeks*:

1. Little interest or pleasure in doing things
2. Feeling down, depressed, or hopeless
3. Trouble falling/staying asleep or sleeping too much
4. Feeling tired or having little energy
5. Poor appetite or overeating
6. Feeling bad about yourself or that you are a failure
7. Trouble concentrating on things (e.g., reading, watching TV)
8. Moving or speaking slowly, or being fidgety/restless
9. Thoughts of being better off dead or hurting yourself

*Scoring*:

- 0 = Not at all, 1 = Several days, 2 = More than half the days, 3 = Nearly every day
- Total score: 0–27

*Recovery Criteria*:

- PHQ-9 score ≤4 (minimal symptoms) sustained for ≥4 weeks.
- PLUS functional improvement (see WHODAS below).

**B. Functional Status (WHODAS 2.0 Short Form)**:

1. Overall daily functioning compared to before depression (same, better, worse).
2. Ability to perform daily activities (0–4 Likert: 0=None, 4=Severe difficulty).
3. Social participation (e.g., community activities; 0–4 Likert).
4. Work/school performance (0–4 Likert).

*Functional Improvement*:

- ≥50% reduction in WHODAS score from baseline OR return to pre-morbid function.

**Final Recovery Determination**:

- Criteria met: (a) PHQ-9 score ≤4 for ≥4 weeks; (b) Patient/family and clinician rate WHODAS items as “no/minimal” impairment.
- If met, check “Recovered.” If not, continue tracking at next visit.

**Documentation**:

- Date: _____________
- Clinician Signature: _____________

**12.0 Supplementary Figure S1: Detailed Epilepsy and Psychosis Clinical Pathways**


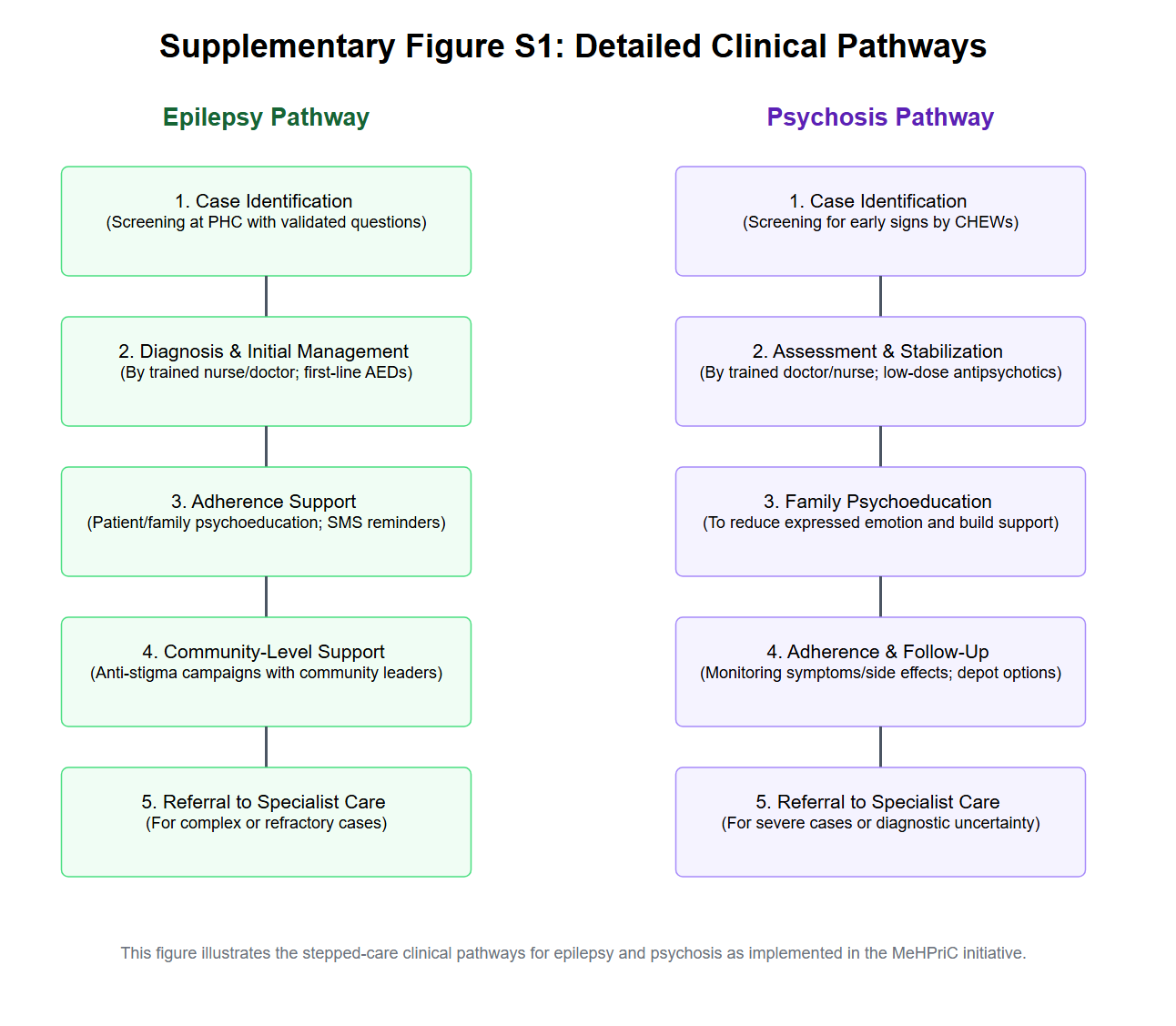

Supplement: Supplementary file 1 — Supplementary Material 1. [file 43058_2025_798_MOESM1_ESM.docx]
